# Editorial: Diffusion-weighted imaging: advances and implementations in neurology

**DOI:** 10.3389/fneur.2026.1878204

**Published:** 2026-05-28

**Authors:** Daniele Corbo, Kevin M. Koch, Kyle C. Kern

**Affiliations:** 1 Department of Medical and Surgical Specialties, Radiological Sciences and Public Health, University of Brescia, Brescia, Italy; 2 Department of Radiology and Imaging, Hospital for Special Surgery, New York, NY, United States; 3 Department of Neurology, UCLA David Geffen School of Medicine, and West Los Angeles VA Medical Center, Los Angeles, CA, United States

**Keywords:** cerebral small vessel disease, CNS tumor, connectome, diffusion tensor imaging, neurodevelopment, spondylotic myelopathy, temporal lobe epilepsy, tractography

Since its introduction, Diffusion-Weighted Imaging (DWI) has evolved into one of the most powerful tools for probing microstructural tissue properties *in vivo*, with applications that now extend far beyond its early neurologic application in identifying cytotoxic edema in acute ischemic stroke. The contributions gathered in this Research Topic highlight how recent methodological advances, quantitative models, and multimodal uses of DWI principles are reshaping our understanding of brain networks, neurodevelopment, and neurological diseases. These translational advances will lead to better clinical trials, more powerful diagnostic and disease monitoring tools, and will ultimately enhance care for patients with neurologic diseases.

Several articles address methodologic improvements in DWI acquisition and standardization. Quantitative DWI metrics are sensitive to a multitude of factors have prevented wider implementation of advanced diffusion models in multi-site research and clinical practice. One major confounding factor is magnetic gradient field inhomogeneity, which introduces systematic spatial errors to quantitative DWI. Krzyzak et al. addressed these errors by correcting for the effective diffusion weighting at each voxel using the B-matrix Spatial Distribution (BSD) method, thereby improving reproducibility. To address commonly encountered site variability in multi-site studies, Pinto et al. validated two harmonization methods, NeuroCombat and LongCombat, in a cohort of 38 healthy participants who were scanned on multiple scanners on the same day. While the original non-harmonized scans demonstrated significant intra- and inter-scanner variability in diffusion metrics, these harmonization methods effectively reducing variance at the voxel and region-of-interest levels. Such harmonization algorithms should be considered when planning multi-site trials using diffusion metrics as outcomes. Finally, Koch et al. implemented multi-spectral metal artifact suppression to improve DWI signal in the spinal cord for patients instrumented with spinal hardware to treat cervical spondylotic myelopathy. Spinal decompression with or without instrumented spinal fusion is a mainstay of treatment for this population. However, post-instrumentation metal artifact hinders subsequent MRI

assessment, DWI in particular, complicating monitoring of disease progression. The authors recruited 38 patients with cervical spondylotic myelopathy after instrumentation and 25 controls to undergo diffusion-weighted multi-spectral imaging (DW-MSI) for artifact suppression. The study showed feasibility in this approach, demonstrating group differences in diffusion metrics globally and within instrumented spinal levels.

Other advancements in diffusion imaging come from refined modeling of the diffusion signal to replicate heterogeneous tissue microstructure. From tensor-based metrics to advanced microstructural estimators, these models enhance sensitivity to subtle pathological changes across the spectrum of neurological disease. Multiple sclerosis (MS) is a prime example of how distinguishing microstructural properties could lead to earlier diagnosis. MS-related disability is driven by diffuse inflammatory mechanisms, but conventional imaging may reveal only subtle findings. DWI, on the other hand, can detect subtle spatially diffuse microstructural changes indicative of neuroinflammation. Mazur-Rosmus et al. combined fractal dimension with diffusion tensor imaging (DTI) metrics to classify MS diagnosis and demonstrate improved classification accuracy over conventional DTI metrics alone. Krzyzak et al. investigated MS diagnostic classification accuracy using a multi-shell DTI protocol with BSD correction for magnetic field inhomogeneity. BSD correction improved the sensitivity of DTI metrics to distinguish diagnosis, particularly when using smaller regions-of-interest that are more susceptible to systematic error.

For central nervous system (CNS) tumors, distinguishing tumor type and grade is critical to treatment. Given the risks of neurosurgical biopsy, biomarkers to non-invasively diagnose or grade tumors are sorely needed. Zhao, Ma et al. demonstrated that DTI- and NODDI-based histogram analysis can effectively distinguish atypical high-grade glioma from primary CNS lymphoma, with the 75th percentile of the orientation dispersion index (ODI) emerging as the most powerful and reliable diagnostic marker. This distinction is critical since CNS lymphoma can be treated medically, while maximal surgical resection is favored for high-grade glioma. A meta-analysis performed by Zhao, Hou et al. illustrated that DKI-derived mean kurtosis and mean diffusivity hold strong promise for non-invasively predicting glioma molecular status, particularly isocitrate dehydrogenase mutation, a marker critical to prognosis. This study demonstrates that imaging-parameter heterogeneity can shape diagnostic performance and motivates the need for robust, standardized MRI-based biomarkers.

DTI tractography is well-known to have utility in neurosurgical planning to avoid injuring eloquent white matter pathways during tumor resection. However, tractography analysis can also predict disability. Chen, Wang et al. showed that tractography in combination with advanced diffusion models (including DTI, DKI, NODDI, and MAP-MRI) effectively detect corticospinal tract injury in patients with glioblastoma. In this study DKI (derived mean kurtosis) provided the most balanced and clinically meaningful sensitivity and specificity profile to support surgical planning and motor-function preservation. This motivates the need for refinement and standardization of tractography approaches to guide neurosurgical centers. Kierońska-Siwak et al. showed that the Frontal Aslant Tract can be reliably delineated using different tractography strategies, with the Superior Frontal Gyrus–pars opercularis pathway providing the most robust reconstruction. They also demonstrated consistent left-hemisphere dominance and age-related microstructural differences in the Frontal Aslant Tract. By integrating these quantitative parameters into intraoperative navigation systems, surgeons can dynamically adjust resection margins to preserve functionally critical fibers and reduce iatrogenic motor deficits.

DWI has also advanced our understanding of cerebral small vessel disease (CSVD), an underrecognized condition that contributes to age related memory decline, vascular cognitive impairment and dementia (VCID), and both ischemic and hemorrhagic stroke. Emerging evidence reviewed by Bozzetti et al. highlight how neurodegenerative mechanisms may be partly driven by endothelial dysfunction, the neurovascular unit, and fluid homeostasis in intravascular, perivascular, and interstitial tissue spaces. This review focuses on the several neuroimaging biomarkers and techniques that have been used to link cerebrovascular dysfunction and neurodegeneration, including advanced diffusion models, tractography, connectomics, voxel-wise and network based analyses, DTI free water (FW) imaging, intravoxel incoherent motion (IVIM), perivascular space (PVS) volumetrics, and the DTI analysis along the perivascular spaces index (DTI-ALPS).

Several articles in this Research Topic demonstrate the utility of DWI in investigating the themes outlined by Bozzetti et al. While hypertensive arteriopathy is the most common contributor to CSVD, hypertension in combination with elevated serum homocysteine, known as H-type hypertension, is associated with even higher risk for stroke, cardiovascular disease and chronic kidney disease. Zhang et al. demonstrated that the DTI-ALPS index is reduced in patients with H-type hypertension, and that this index mediates an association between elevated homocysteine and cognitive performance. DTI-ALPS has been proposed as a surrogate for glymphatic dysfunction (1), although the specificity of this marker for glymphatic activity is uncertain (2, 3). More directly, the work by Zhang et al. supports the utility of DTI in measuring white matter interstitial fluid changes as an important marker for VCID.

DTI-FW is another marker of interstitial fluid changes that is sensitive to CSVD. Kern et al. evaluated white matter DTI-FW changes in patients with prior stroke and white matter hyperintensities, an established marker of CSVD severity. On a voxel-wise level, DTI-FW was associated with growth of white matter hyperintensities over the subsequent year, suggesting that increased interstitial fluid is an early driver of CSVD progression. Feng et al. also investigated the role of interstitial fluid via DTI-FW in CSVD. In their study, enlarged PVS were visually graded in the basal ganglia. Enlarged PVS are a surrogate for glymphatic function, thought to represent stagnant perivascular flow. Their enlargement in the basal ganglia is associated with longstanding hypertension and CSVD severity. The authors found that severely enlarged basal ganglia PVS were linked to greater white matter hyperintensity burden, with DTI-FW emerging as a key mediator connecting impaired perivascular and interstitial fluid drainage to CSVD severity.

Bergamino et al. also used DTI-FW, in addition to NODDI, to investigate white matter alterations, this time in the setting of Parkinson's Disease (PD). Levodopa is a short-acting treatment for PD, leading to fluctuations in control of motor symptoms that can be classified as on vs. off state. In a cohort of 20 patients with PD using levodopa and 16 healthy controls, they used a voxel-wise approach to show that in the off state, patients with PD had altered diffusion metrics in key motor pathways relative to healthy controls, including increased DTI-FW, reduced neurite density index (NDI), and altered ODI. In the on-treatment state, FW and NDI were relatively increased in several pathways compared to off-treatment. Furthermore, diffusion alterations were associated with motor symptoms. These findings suggest that diffusion metrics are sensitive to PD severity and can be dynamic with levodopa treatment.

For ischemic stroke, DWI is the gold standard for diagnosis of acute ischemia and can help to age recent infarcts based on diffusion properties. Though DTI has had less clinical impact on stroke management, it is used frequently deployed in stroke research investigations. In a bibliometric analysis of stroke literature, Chen, Liu et al. found growing scientific interest in DTI applications for ischemic stroke. Through their comprehensive analysis, they mapped global research trends and emerging hotspots, and they identified a need for stronger international collaboration and higher-quality studies to advance the field.

DWI and other neuroimaging modalities have also advanced our understanding of structural and functional brain networks to improve diagnosis and management for patients with epilepsy. When recurrent seizures emanate from an ictal focus, known as focal onset epilepsy, changes in brain connectivity occur that may reflect progressive injury, kindling, and increasing seizure propensity. Qu et al. integrated voxel-wise diffusion metrics with cortical morphometry, structural connectivity, and functional connectivity to characterize network and structural changes related to right temporal lobe epilepsy.

Progress in DWI research continues to elucidate details of healthy brain connectivity and development. Li et al. characterized the structural connectome architecture of medial temporal lobe subregions, identifying distinct network profiles and major connectivity subtypes that reflect their divergent anatomical organization and functional roles. A study by Nayak et al. stands out for its wide developmental coverage, from infancy to early adulthood, and for its innovative combination of volumetric and DTI based metrics enabled by robust diffeomorphic registration across ages. This work provides valuable age-normalized benchmarks and highlights the need for larger longitudinal datasets to refine developmental trajectories.

The contributions in this Research Topic demonstrate the versatility of DWI from microstructural biomarkers and fractal descriptors to network-level analyses and its growing impact on clinical Neurology. By bridging advanced modeling, multimodal integration, and disease-focused applications, these studies collectively highlight both the current achievements and the future potential of diffusion MRI to improve diagnosis, monitoring, and mechanistic understanding of neurological disorders.
